# A novel combined targeted therapy with bromodomain antagonist and MEK inhibitor in anaplastic thyroid cancer

**DOI:** 10.18632/oncotarget.26591

**Published:** 2019-01-22

**Authors:** Carmelo Nucera

**Affiliations:** Carmelo Nucera: Human Thyroid Cancers Preclinical and Translational Research Program, Harvard Medical School, Boston, MA, USA

**Keywords:** anaplastic thyroid cancer, bromodomain and extra-terminal (BET) inhibitors, MEK, BRAFV600E, combined therapy

Anaplastic thyroid cancer (ATC) is the most aggressive thyroid cancer and one of the most lethal of all cancers, as no effective treatment is available. Recently the FDA approved a combination therapy with dabrafenib and trametinib (targeting BRAF^V600E^ and MEK1/2, respectively) for unresectable or metastatic BRAF^V600E^–positive ATC [1]. But there are few if any treatment options available for patients who lack the *BRAF*V600E mutation. Thus new therapeutic strategies against ATC are urgently needed.

The transcription factor Myc propels the progression of ATC, and is often elevated in anaplastic tumor tissues; yet we have no method of directly modulating its activity. Recent studies have shown, however, that bromodomain and extra-terminal (BET) inhibitors can selectively suppress *Myc* transcription by interfering with the interactions between acetylated histones and BET family proteins [2]. Bromodomains are conserved protein modular domains, found in many chromatin- and transcription-associated proteins, that can recognize acetylated lysine residues. These domains are transcriptional co-activators essential for cell cycle regulation, transcriptional activation, and elongation [2, 3]. The bromodomains of the BET family (BRD2, BRD3, BRD4 and BRDT) are critical mediators of chromatin-dependent signal transduction from master regulatory transcription factors to RNA Polymerase II. BRD4 in particular is a co-activator of Myc, and therefore a promising therapeutic target in cancer [2] [4].

In a recent study of a *Thrb*PV/PV*Kras*G12D mouse model of advanced thyroid cancer, BET inhibitor JQ1 was shown to reduce tumor growth, downregulate *Myc* expression [5] and inhibit proliferation of human ATC cell lines harboring different oncogenic drivers. These findings suggest that targeting Myc via BET inhibitors may prove effective in the treatment of ATC.

A recent additional study by Zhu *et al.* [6] assessed the combination of JQ1 with trametinib as a novel therapeutic strategy against ATC. The authors found this combination down-regulated *Myc* expression, and inhibited tumor cell proliferation more effectively than single agents, likely by downregulating levels of pro-survival regulators and up-regulating pro-apoptotic regulators. Importantly, this combined treatment was found to significantly inhibit tumor growth in xenograft mouse models of ATC. The therapeutic success of synergistic suppression of Myc transcription via chromatin modification suggests that combinations of epigenetic modifications and inhibition of MEK1/2 intracellular signaling could lead to new treatment options for ATC.

Indeed, it has emerged from other recent research that a wide range of aberrant mutational signaling can affect transcriptional events in ATC [7, 8]. Thus targeting additional epigenetic modulators of chromatin or other aspects of the general transcription machinery could yield a variety of effective new therapies dependent on a diverse array of mechanisms, or even secondary mechanisms. In Zhu *et al.* [6], for instance, BET and MEK inhibitors blocked the interaction of BET proteins with the promoter of the *Myc* gene to effectively suppress its expression; it is believed that lower Myc expression levels promoted downstream events that in turn triggered apoptosis, which reduced proliferation and inhibited tumor growth.

There is a growing consensus that similar combination treatments may represent a crucial new avenue in oncology, and may have the potential to arrest tumor growth, induce tumor cell death, and even prevent resistance to targeted therapies and immune therapies. However, since ATC harbors a wide variety of genetic alterations affecting proto-oncogenes and tumor suppressors, a thorough genomic characterization of specific types of ATC tumors will be fundamental to developing valid rationales for patient treatment. Preclinical validations for toxicity, therapeutic efficacy and safety will be necessary for any type of combined therapy, and should provide valuable insights into the synergy or antagonism between the combined drugs.

Further analysis of the durable combined action of BET and an MEK inhibitors will be essential to unraveling the hidden mechanisms of drug resistance (both primary and secondary) in ATC, and could serve as an interesting model for exploring aspects of clonal evolution and survival, as described for the first time in a recent study using advanced papillary thyroid carcinoma (PTC)-derived cells that became resistant to vemurafenib, a FDA-approved BRAF^V600E^ inhibitor, via chr.5 aberrations and RBM (RNA-binding motif) family genes mutations [9]. The authors of this study used a combined therapy targeting BRAF^V600E^ with vemurafenib and CDK4/6 with palbociclib to suppress the selection and expansion of aggressive thyroid tumor clones.

Further molecular and structural studies of chromatin complexes based on genes marked by enhancers or super-enhancers will no doubt identify unrecognized ATC dependencies and vulnerabilities, and hence new therapeutic strategies. Subsequent translational studies can then be performed on comprehensive cohorts of ATC patients to accurately evaluate the effects of BET and other inhibitors, and establish therapeutic rationales for this deadly disease.
